# Central Precocious Puberty in a Male Child With Down Syndrome: A Case Report

**DOI:** 10.7759/cureus.68195

**Published:** 2024-08-30

**Authors:** Khalid Al Noaim

**Affiliations:** 1 Department of Pediatrics, College of Medicine, King Faisal University, Al-Ahsa, SAU

**Keywords:** gnrh therapy, trisomy 21, gonadal dysfunction, precocious puberty, down syndrome

## Abstract

Gonadal dysfunction is a well-known endocrine manifestation of Down syndrome (DS) in men. Herein, we report a case of a seven-year-old boy with DS who developed precocious puberty, presenting with a six-month history of frequent erections, sudden growth spurts, and adult odors. Clinical examination revealed stage 2 puberty, with pubic hair development at stage 2 based on Tanner staging and bilateral testicular volumes of 6 mm. Laboratory tests indicated elevated luteinizing hormone (LH) and follicle-stimulating hormone levels and advanced bone age. A pituitary MRI revealed normal pituitary morphology without any detectable masses. The patient was initially prescribed monthly triptorelin acetate (Decapeptyl) intramuscular injections at a dose of 3.75 mg. Following six months, the treatment was switched to triptorelin pamoate (Diphereline) at a dose of 11.25 mg administered intramuscularly every three months. At age 10, following a three-year course of gonadotropin-releasing hormone (GnRH) agonist therapy, the patient demonstrated a notable decrease in testicular size and a reduction in LH levels, with no reported side effects. Conclusively, our findings imply that idiopathic central precocious puberty in boys with DS can be effectively managed with triptorelin pamoate injections, and early intervention may help alleviate the psychosocial impact of this condition.

## Introduction

Children with Down syndrome (DS) have a higher likelihood of acquiring endocrine illnesses such as thyroid dysfunction, diabetes mellitus, short stature, gonadal dysfunction, vitamin D inadequacy, and obesity than the general population [1].

Precocious puberty is defined as the occurrence of puberty-related changes at an age that is more than 2.5 standard deviations below the average. This refers to the occurrence of puberty before the age of eight years in girls and nine years in boys [2]. If precocious puberty is caused by the early activation of the hypothalamic-pituitary-gonadal (HPG) axis and sex hormones are produced by the maturing gonads, it is called central precocious precocity. In males, the testes often undergo enlargement owing to gonadotropin stimulation before any other signs of puberty become apparent. In females, the initial indicators of puberty include breast development and/or pubic hair growth. In central precocious puberty, gonadotropin and gonadal steroid concentrations increase to the typical range observed during puberty [3].

Reduced gonadal function is a widely recognized characteristic of DS [4]. Earlier studies have shown that pubertal males with DS may experience hypergonadotropic hypogonadism, which is defined by elevated levels of follicle-stimulating hormone (FSH) and luteinizing hormone (LH) with low total testosterone levels at the periods of normal puberty, which continues to develop from late puberty to adulthood. Failure of both the Leydig and Sertoli cells is responsible for this condition [5]. Additionally, research has indicated that the Leydig cell dysfunction could be caused by the presence of an extra copy of genetic material from chromosome 21 [6]. Research has revealed that boys with DS typically experience the onset of puberty at an age similar to that of their non-affected peers [7]. Contradictorily, in this study, we describe a boy with DS who exhibited central precocious puberty at the age of seven years.

## Case presentation

**Figure 1:**
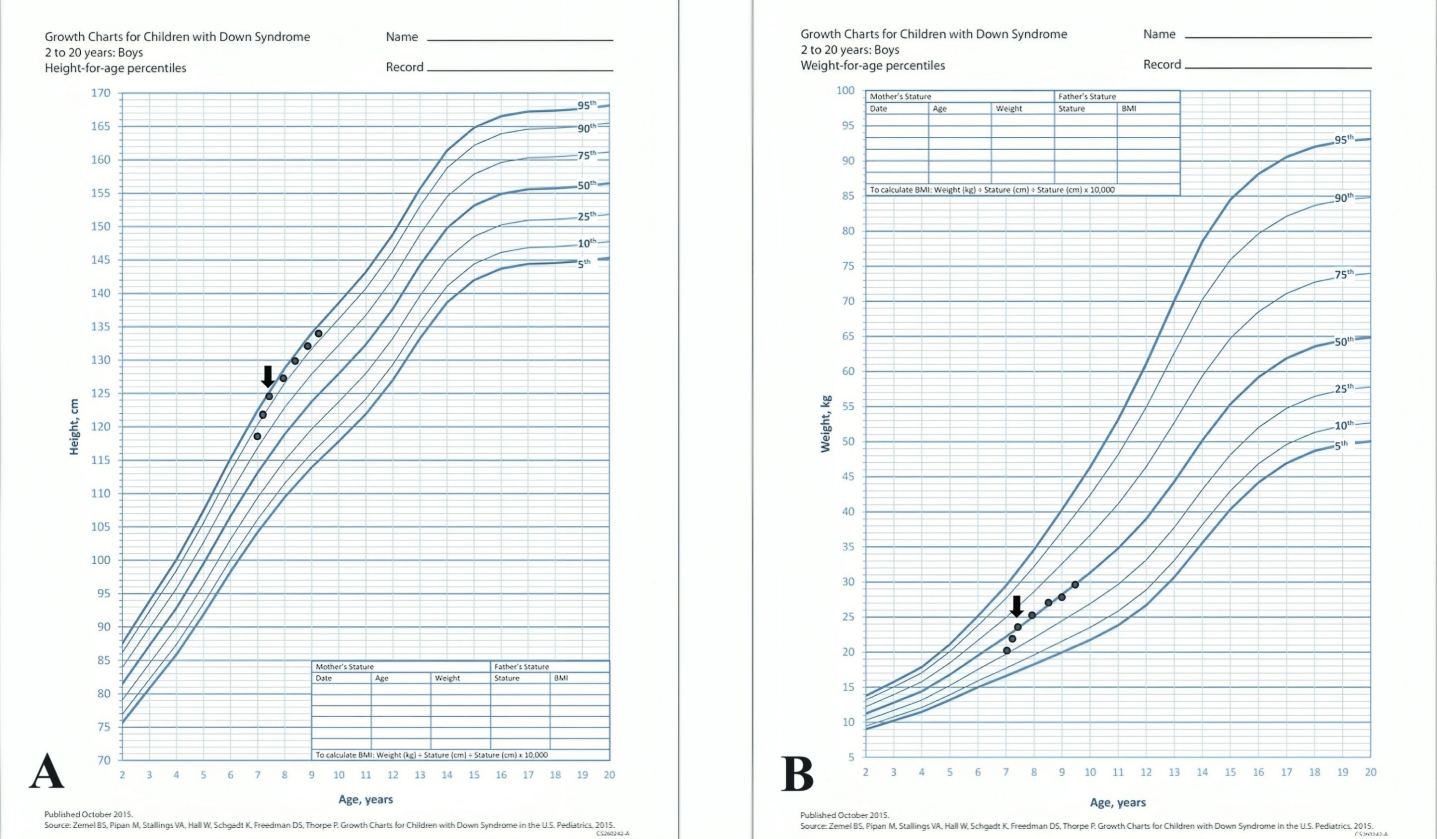
The growth charts showed an acceleration of height gain (A) and an increase in weight (B) at the age of seven years. (A) Down syndrome height for age growth chart. (B) Down syndrome weight for age growth chart. Arrows indicate the time of presentation to starting the treatment.

A seven-year-old boy was diagnosed with DS. He was followed up at another clinic because of acquired hypothyroidism, which was well controlled with levothyroxine. He visited our pediatric endocrine clinic because of symptoms of puberty that had persisted for six months. The child's parents stated that they had begun experiencing frequent erections, a considerable increase in growth, and the development of adult smells. There was no documented record of any symptoms related to the central nervous system or any exposure to drugs known to cause the early onset of puberty. During the physical examination, his height was 124.7 cm, and his weight was 23.2 kg on the 95th and 50th percentage, respectively, in DS growth charts (Figure 1).

Furthermore, the child exhibited pubic hair development at stage 2 based on Tanner staging and bilateral testicular volumes of 6 mm. No detectable lumps or abnormalities were found during the examination of the genitals, and the central nervous system was normal. Laboratory tests showed normal thyroid function, but there was an increase in both baseline and peak LH and FSH levels (Table 1).

**Table 1:** Result of investigations of the presented case. DHEA-S: dehydroepiandrosterone sulfate; FSH: follicle-stimulating hormone; LH: luteinizing hormone; TSH: thyroid-stimulating hormone.

Test	Reference ranges for age	Result
Baseline LH (mIU/ml)	0.02-0.3	0.32
Baseline FSH (mIU/ml)	0.28-3	0.76
Stimulated LH (mIU/ml)	<2.8	7.9
Stimulated FSH (mIU/ml)	-	8.9
Testosterone (ng/dl)	<0.2-1.3	<0.45
DHEA-S (mcg/dl)	<72	72
17-OH progesterone (ng/ml)	<0.9	1.1
TSH (mIU/ml)	0.6-5.5	3.5
Free T4 (ng/dl)	0.9-1.67	1.32

**Figure 2:**
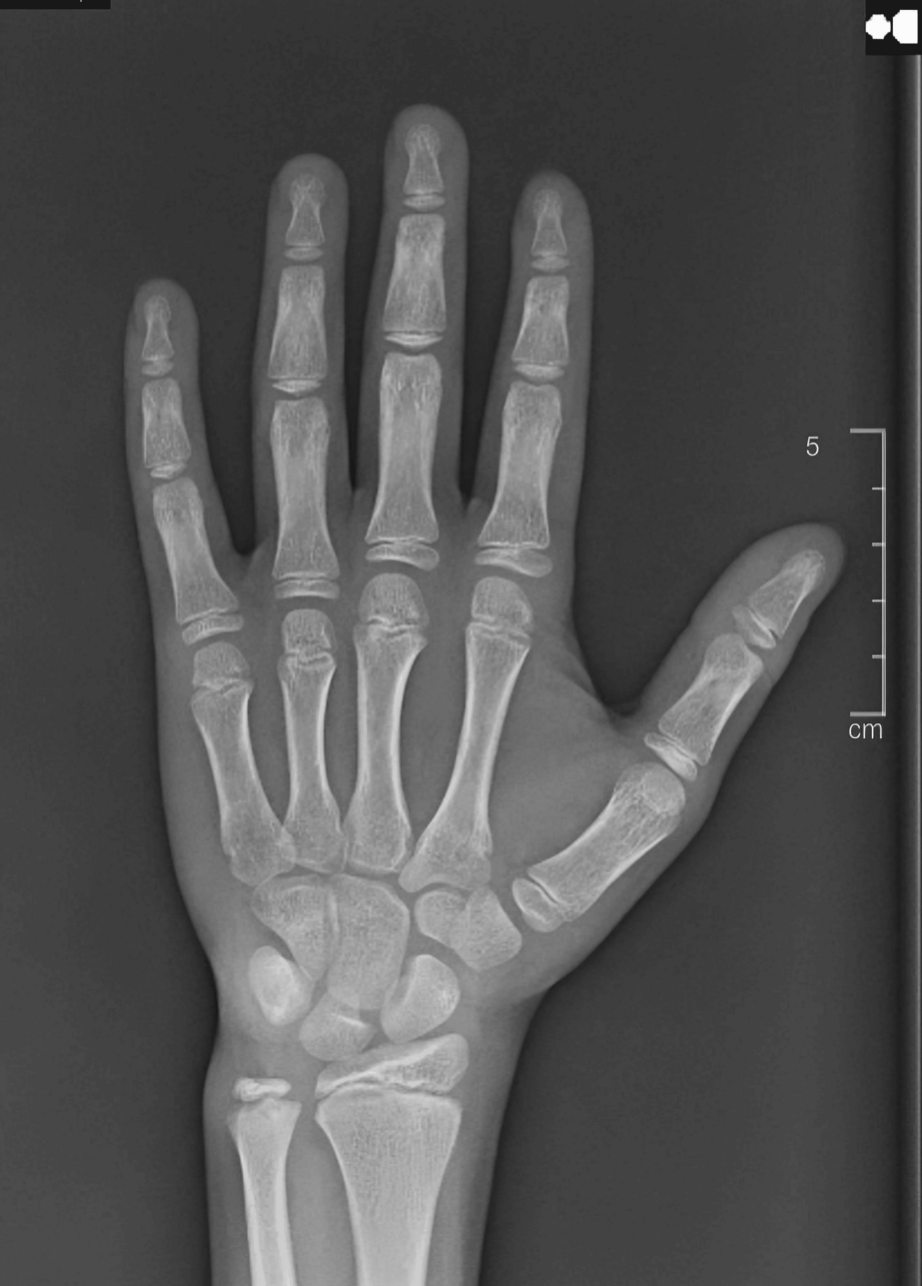
X-ray of the left hand showed a bone age corresponding to 10 years.

**Figure 3:**
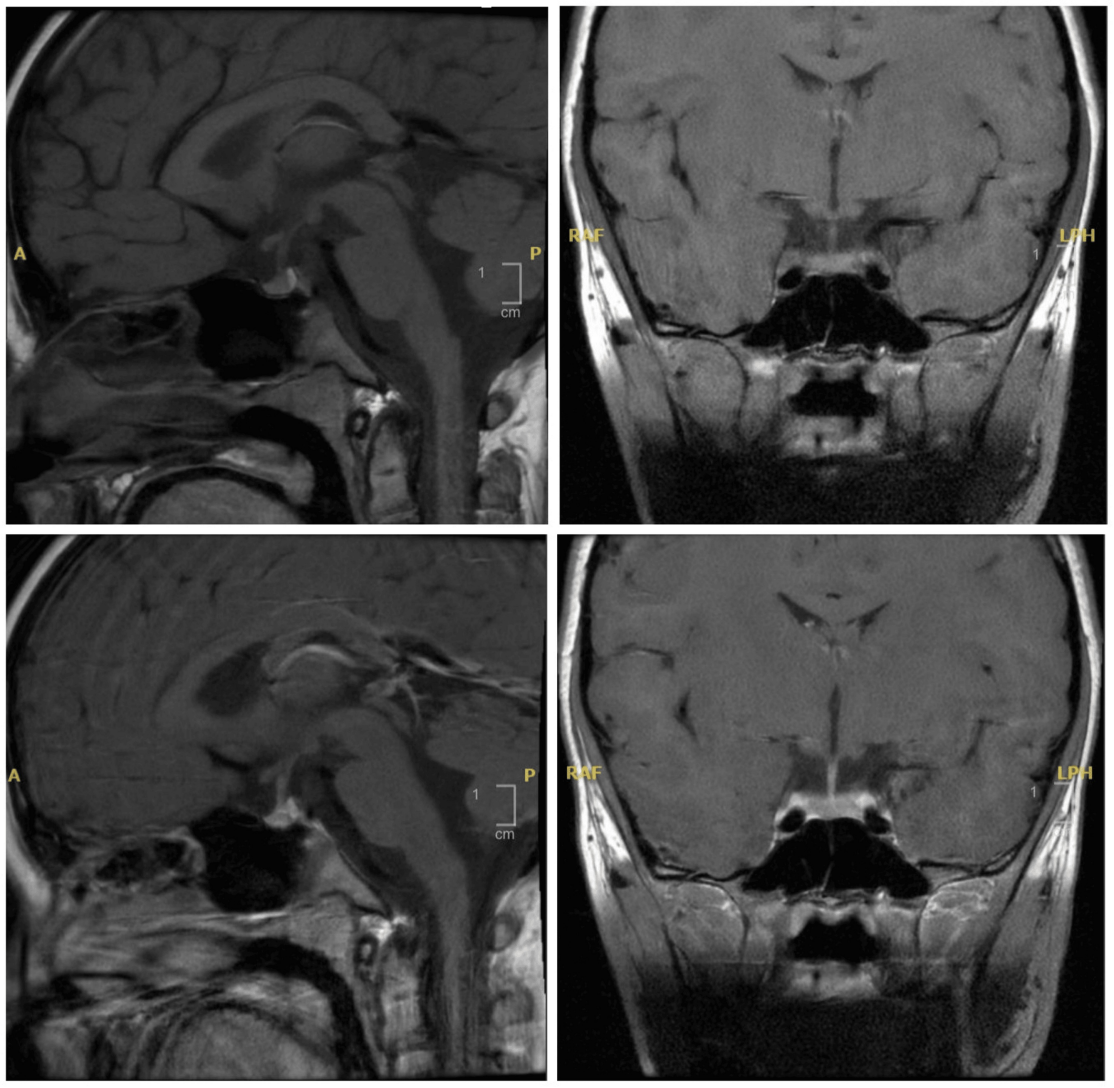
Pituitary MRI showed normal pituitary gland morphology and centralized pituitary stalk.

Radiological analysis revealed accelerated bone development, with a bone age of 10 and a chronological age of seven (Figure 2). The pituitary MRI indicated a normal pituitary gland without any central nervous system masses or pituitary abnormalities (Figure 3).

The patient was initially prescribed triptorelin acetate (Decapeptyl) at a monthly intramuscular dose of 3.75 mg. Subsequent laboratory tests consistently showed highly favorable results with LH suppression and clinical improvement in the form of a reduction in the rate of growth and static testicular size (Figure 1). After six months, the child was transitioned to triptorelin pamoate (Diphereline) at a dosage of 11.25 mg administered intramuscularly every three months. The child is currently 10 years old and has successfully completed a three-year treatment with a gonadotropin-releasing hormone (GnRH) agonist, showing a great response in the form of a decrease in testicular size and a low LH level without identified side effects.

## Discussion

Gonadal dysfunction is a hormonal condition documented in males with DS [4]. Central precocious puberty has rarely been observed in male children with DS. Our patient showed idiopathic central precocious puberty, in which the boy had enlarged testes, increased peak LH levels, advanced bone maturation, and a normal pituitary MRI scan. To date, only two cases have been reported in depth involving boys with DS experiencing central precocious puberty [8]. The two cases started to have signs of puberty at age four and seven years, respectively. These cases showed elevated peak LH, and abnormal MRI results: a small pituitary gland in the first case and periventricular millimetric foci in the supratentorial region in the second case. Both cases were treated with leuprolide acetate monthly intramuscular injections [8]. This is the first report of a case of idiopathic central precocious puberty in a male patient with DS who exhibited normal brain MRI and the first case report that explained the response to monthly triptorelin acetate and three-monthly triptorelin pamoate in DS with central precocious puberty.

Precocious puberty in males with DS is likely to be influenced by genetic factors or the presence of other medical problems, such as untreated primary hypothyroidism, which might lead to Van Wyk-Grumbach syndrome [9]. The presented case had hypothyroidism but it was well controlled in levothyroxine as represented by normal thyroid function during routine follow-up. Furthermore, LH levels in Van Wyk-Grumbach syndrome will be suppressed because the primary influence of precocious puberty in these cases is the high TSH level, which is not the case in this patient [9].

Researchers have found that DS can cause variations in the timing of puberty and disease progression. Cases of DS are believed to have the same onset of puberty as their peers but reach the last stage of puberty later than their peers [10]. The mechanism underlying hormonal alterations is poorly understood; however, some researchers have indicated that changes in the HPG axis or gonadal function may be contributing factors [6]. Adolescent boys with DS undergo physical transformations; some experience unusual growth spurts, changes in body shape, and the development of secondary sex features [11].

For males with DS, puberty usually had delayed completion. It sometimes showed a unique pubertal growth spurt as the onset was usually earlier by one year. Their peak height velocity was about 1.3 to 1.4 cm shorter than their peers, with retardations in skeletal maturation [11]. Furthermore, individuals with DS may experience difficulties in weight control and adaptation to changes in body composition during puberty, making them prone to obesity and health-related complications [6]. Additionally, obesity in children with DS may be associated with elevated aromatase activity, which converts testosterone into estradiol [12].

Young boys with DS are at risk of developing cognitive and behavioral disturbances, which may present more difficulties to their families. During puberty, it has been noted that young adults with DS may have increased emotional sensitivity, mood swings, and behavior issues. These behavioral changes might be due to recent changes in hormonal levels in addition to societal factors [13]. Consequently, individuals with DS can experience cessation or even a decline in cognitive development during adolescence. To this end, specific educational and behavioral interventions must be implemented to enhance learning and socialization [13]. A recent study demonstrated the role of GnRH pulsatile therapy in improving cognitive function and brain connectivity in specific brain regions of patients with DS [14].

Males with DS are considered infertile; however, the etiology of infertility is unknown. The suggested causes of infertility include hormonal deficiencies, morphological gonadal defects, abnormal spermatogenesis, and psychosocial factors related to intellectual disabilities. Extra chromosome 21 directly and indirectly affects the reproductive capacity of males with DS. However, the definite cause of insufficient or inadequate spermatogenesis remains unknown [15]. There are some reported cases of DS in men who can achieve fertility, but these are considered extremely rare [16-18].

The previous two cases were treated with leuprolide 3.75 mg or 7.5 mg monthly intramuscular injections [8]. The patient was initially managed with monthly injections of triptorelin acetate. Subsequently, we switched to triptorelin pamoate every three months. Both medications were used for the first time in patients with DS and central precocious puberty and showed an excellent response without any side effects. Previous studies have shown that monthly leuprolide and triptorelin treatments in children with idiopathic central precocious puberty have similar efficacy and tolerability [19]. Another study showed that triptorelin pamoate (injected every three months) provided a more effective and significant reduction in the LH peak after 12 months of treatment in comparison to triptorelin acetate (injected monthly), in addition to the lower injection frequency [20].

## Conclusions

Boys with DS may develop idiopathic central precocious puberty that can be effectively managed with triptorelin pamoate injections. Early intervention may improve the psychosocial impact associated with this condition.

## References

[1] Bull MJ (2020). Down syndrome. N Engl J Med.

[2] Bianco SD (2012). A potential mechanism for the sexual dimorphism in the onset of puberty and incidence of idiopathic central precocious puberty in children: sex-specific kisspeptin as an integrator of puberty signals. Front Endocrinol (Lausanne).

[3] Livadas S, Chrousos GP (2019). Molecular and environmental mechanisms regulating puberty initiation: an integrated approach. Front Endocrinol (Lausanne).

[4] Hasen J, Boyar RM, Shapiro LR (1980). Gonadal function in trisomy 21. Horm Res.

[5] Grinspon RP, Bedecarrás P, Ballerini MG (2011). Early onset of primary hypogonadism revealed by serum anti-Müllerian hormone determination during infancy and childhood in trisomy 21. Int J Androl.

[6] Attia AM, El Naqeeb HH, Ghanayem NM (2015). Sexual and reproductive functions in men with Down′s syndrome. Menoufia Med J.

[7] Hsiang YH, Berkovitz GD, Bland GL, Migeon CJ, Warren AC (1987). Gonadal function in patients with Down syndrome. Am J Med Genet.

[8] Guven A, Cebeci AN (2020). A rare endocrine manifestation of Down syndrome: central precocious puberty: three cases report. Eur J Med Case Rep.

[9] Lim HH, Kil HR, Kim JY (2012). Unusual presentations of a girl with Down syndrome: Van Wyk-Grumbach syndrome. J Pediatr Endocrinol Metab.

[10] Erdoğan F, Güven A (2022). Is there a secular trend regarding puberty in children with down syndrome?. Front Endocrinol (Lausanne).

[11] Kimura J, Tachibana K, Imaizumi K, Kurosawa K, Kuroki Y (2003). Longitudinal growth and height velocity of Japanese children with Down's syndrome. Acta Paediatr.

[12] Hestnes A, Stovner LJ, Husøy O, Følling I, Fougner KJ, Sjaastad O (1991). Hormonal and biochemical disturbances in Down's syndrome. J Ment Defic Res.

[13] Grieco J, Pulsifer M, Seligsohn K, Skotko B, Schwartz A (2015). Down syndrome: cognitive and behavioral functioning across the lifespan. Am J Med Genet C Semin Med Genet.

[14] Manfredi-Lozano M, Leysen V, Adamo M (2022). GnRH replacement rescues cognition in Down syndrome. Science.

[15] Stefanidis K, Belitsos P, Fotinos A, Makris N, Loutradis D, Antsaklis A (2011). Causes of infertility in men with Down syndrome. Andrologia.

[16] Sheridan R, Llerena J Jr, Matkins S, Debenham P, Cawood A, Bobrow M (1989). Fertility in a male with trisomy 21. J Med Genet.

[17] Pradhan M, Dalal A, Khan F, Agrawal S (2006). Fertility in men with Down syndrome: a case report. Fertil Steril.

[18] Jazayeri O, Gorjizadeh N (2020). A male Down syndrome with two normal boys: cytogenetic, paternity and andrological investigations. Andrologia.

[19] Valenzise M, Nasso C, Scarfone A (2023). Leuprolide and triptorelin treatment in children with idiopathic central precocious puberty: an efficacy/tolerability comparison study. Front Pediatr.

[20] Lasorella S, Porto R, Iezzi ML, Pistone C, Marseglia GL, Verrotti A, Brambilla I (2020). Comparison of triptorelin acetate vs triptorelin pamoate in the treatment of central precocious puberty (CPP): a retrospective study. Gynecol Endocrinol.

